# Case report: Isolated immunoglobulin G4-related sclerosing cholangitis misdiagnosed as hilar cholangiocarcinoma

**DOI:** 10.3389/fonc.2024.1385214

**Published:** 2024-05-23

**Authors:** Hui Li, Ran Wang, Dongyang Wang, Yufu Tang, Xuantong Liu, Hongyu Li, Xingshun Qi

**Affiliations:** ^1^ Department of Gastroenterology, General Hospital of Northern Theater Command, Shenyang, China; ^2^ Department of Life Sciences and Biopharmaceutics, Shenyang Pharmaceutical University, Shenyang, China; ^3^ Department of Hepatobiliary Surgery, General Hospital of Northern Theater Command, Shenyang, China; ^4^ Department of Pathology, General Hospital of Northern Theater Command, Shenyang, China

**Keywords:** IgG4, surgery, cholangitis, steroids therapy, rituximab

## Abstract

**Background:**

Immunoglobulin G4-related sclerosing cholangitis (IgG4-SC) is frequently accompanied with type 1 autoimmune pancreatitis (AIP). Isolated IgG4-SC which is not accompanied with AIP is uncommon in clinical practice, and its manifestations are similar to those of hilar cholangiocarcinoma.

**Case presentation:**

A 55-year-old male presented with persistent aggravation of icteric sclera and skin. He was initially diagnosed with hilar cholangiocarcinoma and underwent surgery. However, positive IgG4 plasma cells were found in the surgical specimens. Thus, a pathological diagnosis of IgG4-SC was established. After that, steroid therapy was given and initially effective. But he was steroid dependent, and then received rituximab therapy twice. Unfortunately, the response to rituximab therapy was poor.

**Conclusion:**

It is crucial to differentiate isolated IgG4-SC from hilar cholangiocarcinoma to avoid unnecessary surgery. Future studies should further explore effective treatment strategy in patients who do not respond to steroids therapy. It is also required to develop novel and accurate diagnostic approaches to avoid unnecessary surgical procedures.

## 1 Introduction

Immunoglobulin G4-related sclerosing cholangitis (IgG4-SC) is an autoimmune disease characterized by fibroinflammatory lesions and their associated bile duct stricture (1). The prevalence of IgG4-SC was 0.63 per 100,000 population in Japan. However, there is a lack of epidemiological studies for IgG4-SC, especially outside of Japan (2). IgG4-SC frequently accompanied with type 1 autoimmune pancreatitis (AIP). IgG4-SC which is not accompanied with AIP is defined as isolated IgG4-SC (3). Isolated IgG4-SC is uncommon in clinical practice, and its manifestations are similar to those of hilar cholangiocarcinoma. Both of them have imaging evidence of strictures in the hilar bile ducts, accompanied by the clinical manifestation of jaundice. Thus, it is often difficult to differentiate between the two diseases, especially in patients with hilar bile duct stricture associated with jaundice (3). Notably, the primary treatment of hilar cholangiocarcinoma is surgical resection (4). By comparison, according to the international consensus, the first-line treatment of IgG4-SC is steroids therapy (3, 5). Accordingly, it is very necessary to establish a definite diagnosis to avoid unnecessary surgery.

Few cases of isolated IgG4-SC have been reported. Herein, we reported a case of isolated IgG4-SC, which was initially misdiagnosed as hilar cholangiocarcinoma and treated with surgery. In addition, we discussed the diagnosis and treatment strategy of isolated IgG4-SC by reviewing the current evidence.

## 2 Case presentation

**Figure 1:**
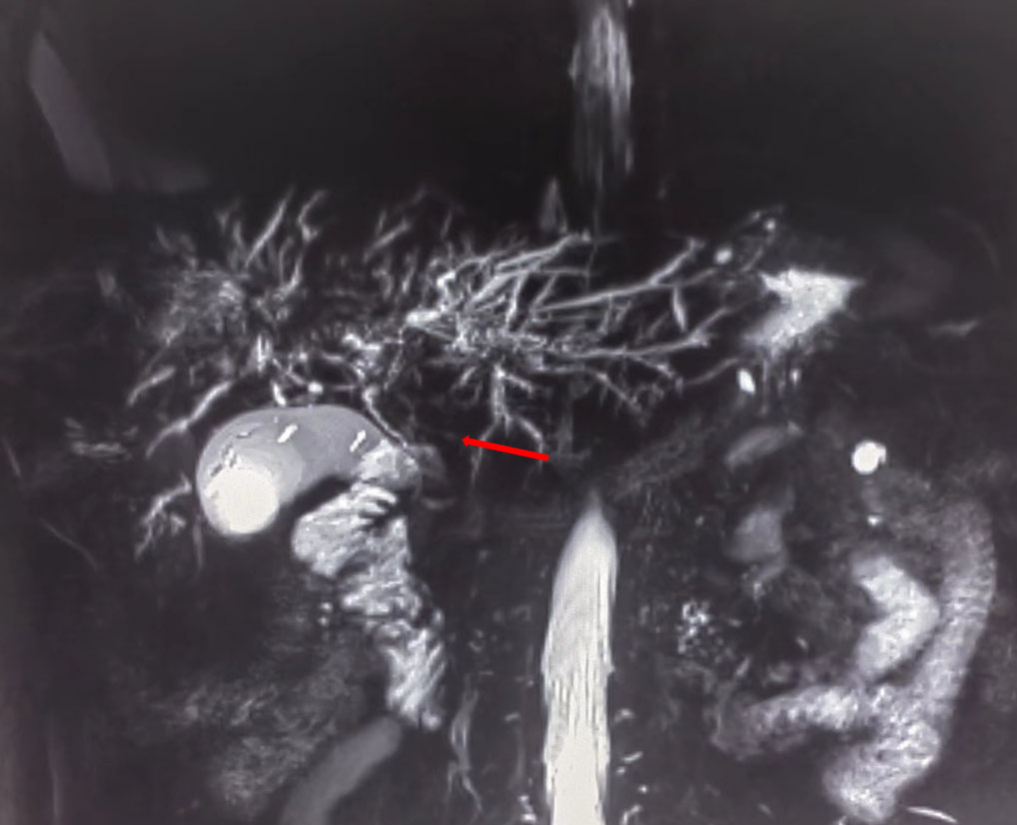
Magnetic resonance cholangiopancreatography (MRCP) showing strictures in the hilar region of the bile duct.

**Figure 2:**
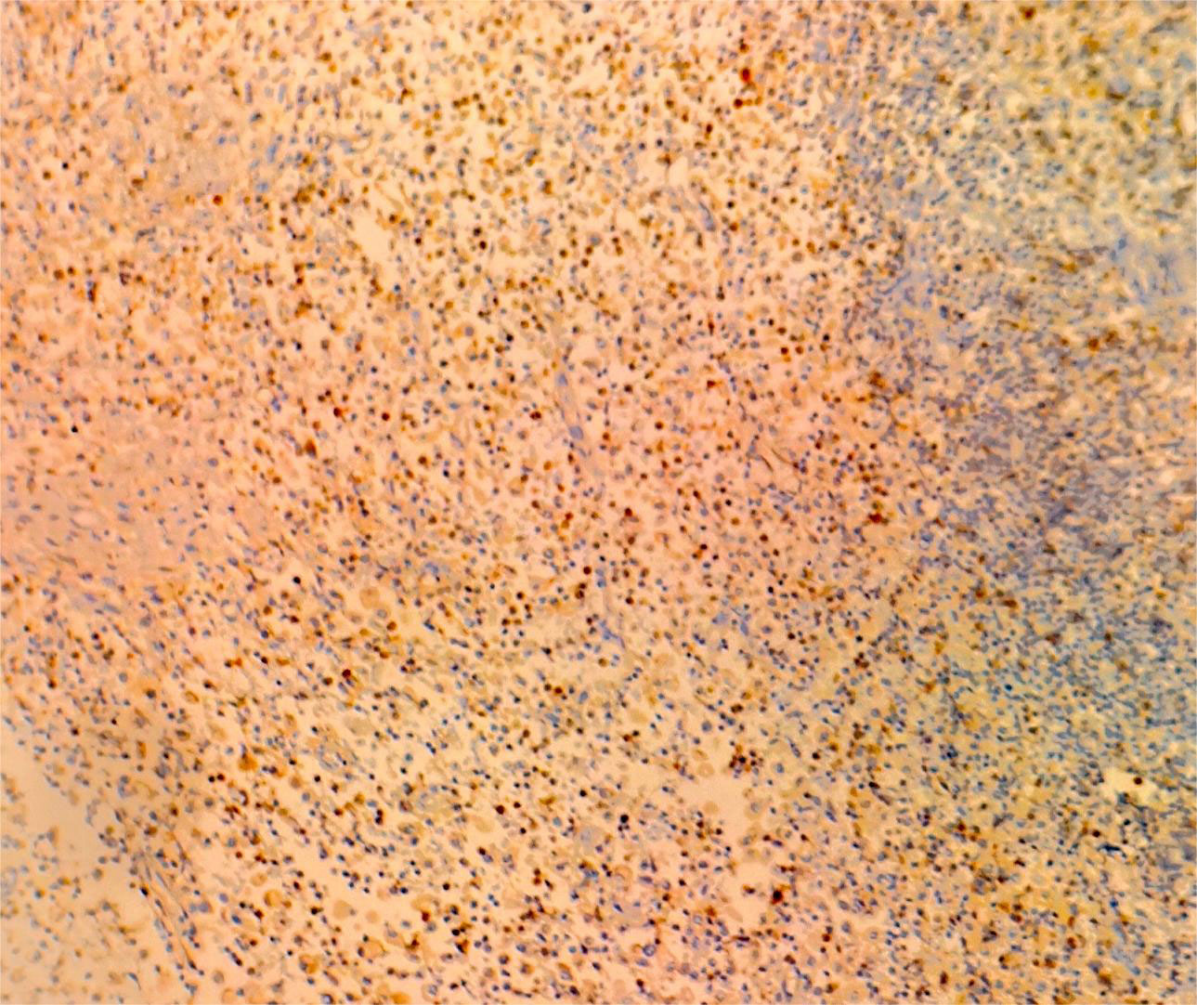
Pathological examination of the surgical specimens in this patient.

**Figure 3:**
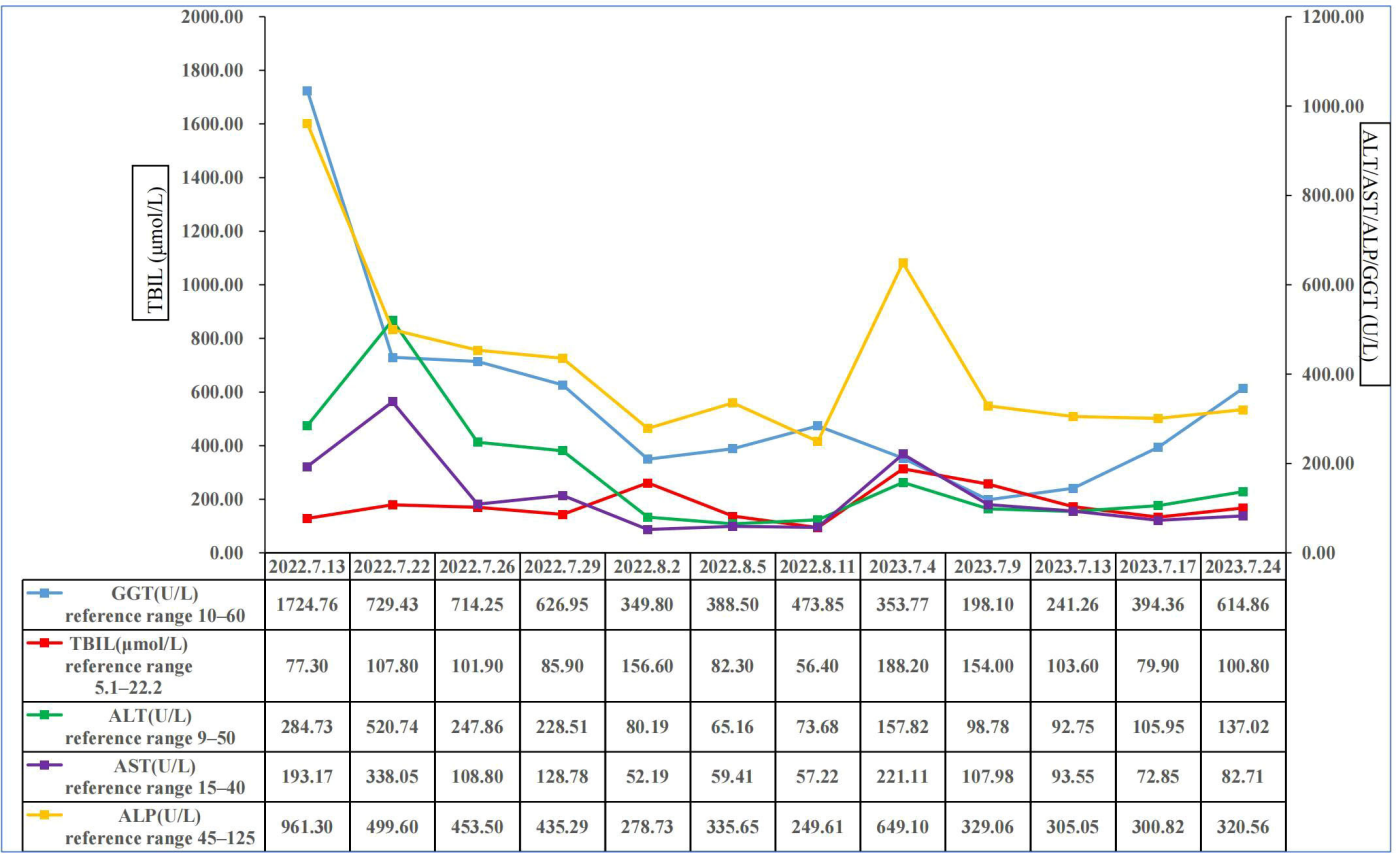
Changes of total bilirubin, alanine aminotransferase, aspartate aminotransferase, alkaline phosphatase, and gamma glutamyl transferase in this patient. ALP, alkaline phosphatase; ALT, alanine aminotransferase; AST, aspartate transaminase; GGT, gamma glutamyl transferase; TBIL, total bilirubin.

On July 13, 2022, a 55-year-old male was admitted to the Department of Gastroenterology due to persistent aggravation of icteric sclera and skin during the past 6 months. The patient has diabetes mellitus and is currently managing blood sugar levels with insulin injections. Laboratory tests revealed elevated levels of total bilirubin (77.3 μmol/L, reference range, 5.1–22.2 μmol/L), direct bilirubin (58.0 μmol/L, reference range, 0–8.6 μmol/L), alkaline phosphatase (961.30 U/L, reference range, 45–125 U/L), gamma glutamyl transferase (1,724.76 U/L, reference range, 10–60 U/L), alanine aminotransferase (284.73 U/L, reference range, 9–50 U/L), aspartate aminotransferase (193.17 U/L, reference range, 15–40 U/L), tumor marker carcinoembryonic antigen 50 (78.68 U/mL, reference range, 0–20 U/mL), and tumor marker carcinoembryonic antigen 199 (100.1 U/mL, reference range, 0–40 U/mL). Hepatitis virus A, B, C, and E were negative. Abdominal ultrasonography showed intrahepatic bile duct dilatation. Contrast-enhanced magnetic resonance imaging (MRI) and magnetic resonance cholangiopancreatography (MRCP) revealed intrahepatic bile duct dilatation and an indistinct image at the confluence of the hilar bile duct, but without evidence of dilatation in the common bile duct or pancreatic duct nor diffuse pancreatic enlargement (**Figure 1**). Primary sclerosing cholangitis was ruled out due to the absence of band-like strictures or a beaded appearance of the biliary tract on MRCP. Following a consultation with surgeons from the Department of Hepatobiliary Surgery, a diagnosis of hilar cholangiocarcinoma could not be excluded. After this patient and his family members signed the written informed consents, left hepatectomy and cholecystectomy with hepatobiliary jejunostomy were performed on July 20, 2022. During the surgery, it was noted that the gallbladder was slightly enlarged with mild edema and thickening of the gallbladder wall. The bile duct was noticeably hardened and narrowed. Upon transverse incision of the bile duct, circumferential thickening of the bile duct wall was observed. No bile flow was observed, indicating bile duct obstruction. The amount of bleeding was 400ml. There was neither intraoperative nor postoperative complications. However, pathological examinations of the surgical specimens did not demonstrate malignant cells, but inflammatory lesions, fibrous tissue hyperplasia, and atypical hyperplasia were observed. Furthermore, immunohistochemical staining was positive for IgG4 plasma cells (>10 per high-power field), leading to a diagnosis of isolated IgG4-related-SC (**Figure 2**). Meanwhile, serum IgG4 level was also measured with a level of 120.31 mg/dL (reference range, 3.92 to 86.4 mg/dL). After that, intravenous methylprednisolone sodium succinate was initiated at a daily dosage of 40 mg for 7 days, which was subsequently transitioned to oral prednisolone acetate tablets at a dosage of 40 mg/day. Gradually, there was notable improvement of liver function and resolution of jaundice (**Figure 3**). The patient was discharged 25 days after the surgery. The dosage of prednisolone acetate tablets was reduced by 5 mg per week, ultimately reaching a maintenance dosage of 5 mg/day.

**Figure 4:**
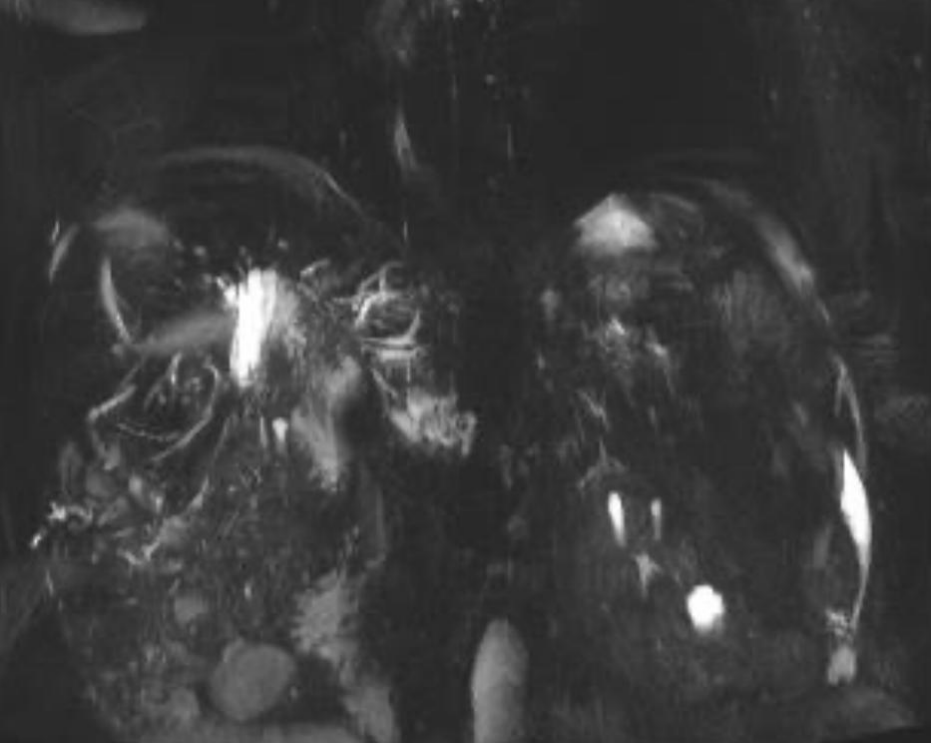
Magnetic resonance cholangiopancreatography (MRCP) showing no indication of anastomotic stricture.

On July 4, 2023, the patient was readmitted to the Department of Gastroenterology due to persistent aggravation of icteric sclera and skin for 2 months. Laboratory tests showed elevated levels of total bilirubin, direct bilirubin, alkaline phosphatase, gamma glutamyl transferase, alanine aminotransferase, and aspartate aminotransferase (**Figure 3**). The results of imaging examination did not indicate anastomotic stricture (**Figure 4**). A relapse of IgG4-SC was considered. A relapse of IgG4-SC is commonly characterized by the reappearance of symptomatic, serologic, radiologic, and/or histological abnormalities following complete or partial remission of the disease (6). The dosage of prednisolone acetate tablets was titrated to 50 mg/day. However, the level of total bilirubin was still abnormal (103.6μmol/L, reference range 5.1 to 22.2 μmol/L). Thus, prednisolone acetate tablets had to be kept at a maintenance dosage of 50 mg/day.

Considering a possibility of steroid dependency in this patient, rituximab therapy was initiated intravenously at 1 g/week twice on August 4, 2023 and August 23, 2023. Prednisolone acetate tablets were maintained at a dosage of 45 mg/day. Despite so, his symptoms did not improve. He refused further intervention and requested to be discharged. Unfortunately, his wife told us that the patient died on November 2023 at the last telephone follow-up visit.

## 3 Discussion

IgG4-SC is predominant in males. Approximately 80% of cases IgG4-SC develop in males (3). IgG4-SC is categorized by its correlation with AIP and the location of bile duct stenosis (7). First, according to its correlation with AIP, IgG4-SC can be categorized into two distinct types: one is associated with AIP, and the other is isolated IgG4-SC in the absence of AIP (3). In a large Japanese cohort study, AIP was identified in 83.7% (730/872) of the cases involving IgG4-SC (8). Furthermore, isolated IgG4-SC was detected in only 8% (9/115) of cases in a prospective UK cohort study (9). Until now, only a few cases with isolated IgG4-SC have been reported, some of whom have undergone unnecessary surgical resection due to their misdiagnoses (10–16). Second, according to the location of bile duct stenosis, IgG4-SC can be categorized into four types: type 1 IgG4-SC, which is characterized by stenosis in the lower bile duct; type 2a, which is characterized by stenosis in the intrahepatic bile duct accompanied with stricture dilatation, whereas type 2b, which is characterized by intrahepatic bile duct stenosis without stricture dilatation and a diminished number of branches in the bile duct; type 3 IgG4-SC, which is distinguished by stenosis in both the hilar region and the lower bile duct; type 4 IgG4-SC, which is distinguished by stenosis exclusively in the hilar region of the bile duct (17). In our patient, type 4 IgG4-SC was considered. According to the results of MRCP, imaging evidence indicates stenosis exclusively in the hilar bile duct, which is consistent with the characteristics of type 4 IgG4-SC. In a prospective Japanese cohort study, type 4 IgG4-SC was identified in only 10% of the IgG4-SC cases (18). As mentioned in the current guideline, it is crucial to differentiate type 4 IgG4-SC from hilar cholangiocarcinoma to avoid unnecessary surgical resection (19) (**Table 1**).

**Table 1:** Differential diagnoses of isolated IgG4-SC and hilar cholangiocarcinoma.

	Isolated IgG4-SC	Hilar cholangiocarcinoma
**Clinical presentation**	Jaundice, pruritus, abdominal pain, and weight loss	Similar symptoms with isolated IgG4-SC, but often a more rapid progression of jaundice and weight loss
**Serum IgG4 level**	Often elevated	Not typically elevated
**Bile ducts (cholangiogram)**	Intrahepatic bile duct dilatation and strictures in the hilar bile ducts	Similar manifestations with isolated IgG4-SC
**Narrowed area of the bile duct**	Concentric symmetrical thickening of the bile duct wall, with smooth inner and outer surfaces and homogeneous internal echoes	Non-concentric thickening of the bile duct wall, with outward concavity of the outer edge and a papillary appearance of the inner edge
**Thickness of the bile duct wall in non-narrowed areas**	>0.8 mm	<0.8 mm
**Response to steroid therapy**	Effective	Not effective

IgG4-SC, Immunoglobulin G4-related sclerosing cholangitis.

Two diagnostic criteria of IgG4-SC are widely accepted: the HISORt criteria (20) and the Japan Biliary Association criteria (17). The HISORt criteria include a combination of five factors: histology, imaging, serology, other organ involvement, and response to steroids therapy (21). The criteria established by the Japan Biliary Association include six elements: narrowing of the intrahepatic and/or extrahepatic bile duct, thickening of the bile duct wall, serological findings, pathological findings, involvement of other organs, and response to steroids therapy (17).

In our case, the imaging revealed hepatic hilar biliary stricture, and the surgical specimens demonstrated circular thickening of the bile duct wall, which was not obvious in CT and MRI scans. There is a superiority of endoscopic ultrasonography and intraductal ultrasonography in providing higher resolution images of the bile duct wall. Thus, they are valuable tools for distinguishing IgG4-SC from other mimickers (21). During the procedures of endoscopic ultrasonography and intraductal ultrasonography, bile duct biopsy and cytology are also clinically significant and necessary to further distinguish IgG4-SC from other diseases (17). Unfortunately, they were not available in our case. Regardless, histological examination of our patient revealed the presence of inflammatory lesions and fibrous tissue hyperplasia without identification of malignant cells as well as positive IgG4 plasma cells >10 per high-power field. Additionally, the serum IgG4 level was 120.31 mg/dL. Notably, according to the diagnostic criteria for IgG4-SC proposed by the Japan Biliary Association criteria, the diagnostic threshold is set at a serum IgG4 level of ≥135 mg/dL (17). However, it has also been reported that patients diagnosed with isolated IgG4-SC may exhibit a serum IgG4 level of <135 mg/dL (12, 14, 22, 23). Moreover, hypergammaglobulinemia, high serum IgG4 concentrations, high serum IgE, high soluble IL-2 receptor, and negative CRP have been reported for IgG4-related disease (24).

The standard treatment for IgG4-SC is steroids therapy (17, 21). The Japanese clinical practice guidelines recommend oral prednisolone at a dosage of 0.6 mg/kg/day for treatment (3). Early-stage IgG4-SC patients respond positively to steroids therapy (18), because inflammation is predominant at this stage. By contrast, advanced-stage IgG4-SC patients may be steroid dependent or resistant, given relatively less inflammation but marked fibrous scars at the advanced stage (25). Initially, our patient had a rapid response to the steroids therapy but experienced the first recurrence after a reduction in the dosage of steroids. Additionally, he was considered to be steroids dependent, because high-dose steroid maintenance therapy had to be employed. In this case, second-line treatment options, including rituximab, a CD20-depletion agent, can be initiated (26, 27). The pathophysiology of IgG4-SC involves the interplay between B and T lymphocytes, resulting in tissue fibrosis and organ damage. Rituximab can induce remission in IgG4-SC by depleting B cells (27). Rituximab is only considered when patients do not respond effectively to steroid treatment. Rituximab has been reported as an effective treatment for patients with IgG4-SC who did not respond well to steroids therapy (27–29). However, in some cases, there was a reduced responsiveness to the effects of rituximab (30). Our patient did not show any positive response to the treatment of rituximab.

## 4 Conclusion

Our case emphasizes the clinical significance of distinguishing between isolated IgG4-SC and cholangiocarcinoma before intervention, especially surgery. To avoid unnecessary surgical resection, it is crucial to integrate the findings from histology, imaging, and serology to establish an accurate diagnosis. In the case of steroid dependency and resistance, clinicians should actively consider second-line therapies for isolated IgG4-SC. Certainly, new treatment strategies will be necessary for patients who do not respond effectively to steroids therapy.

## Data availability statement

The original contributions presented in the study are included in the article/supplementary material. Further inquiries can be directed to the corresponding authors.

## Ethics statement

Written informed consent was obtained from the participant for the publication of this case report.

## Author contributions

HuL: Writing – original draft, Data curation, Investigation, Software, Validation, Writing – review & editing. RW: Writing – review & editing, Data curation, Investigation, Validation. DW: Writing – review & editing, Data curation, Investigation, Validation. YT: Writing – review & editing, Data curation, Investigation, Validation. XL: Writing – review & editing, Data curation, Investigation. HoL: Writing – review & editing, Data curation, Investigation, Supervision, Validation. XQ: Writing – review & editing, Conceptualization, Data curation, Investigation, Supervision, Validation.

## References

[1] Hubers LM Maillette de Buy Wenniger LJ Doorenspleet ME Klarenbeek PL Verheij J Rauws EA . IgG4-associated cholangitis: a comprehensive review. Clin Rev Allergy Immunol. (2015) 48:198–206. doi: 10.1007/s12016-014-8430-2 24958363

[2] Khoury NC Birk JW . A review of igG4-related sclerosing cholangitis (IgG4-SC). J Clin Gastroenterol. (2024). doi: 10.1097/MCG.0000000000001984 38385591

[3] Kamisawa T Nakazawa T Tazuma S Zen Y Tanaka A Ohara H . Clinical practice guidelines for IgG4-related sclerosing cholangitis. J hepato-biliary-pancreatic Sci. (2019) 26:9–42. doi: 10.1002/jhbp.596 PMC6590186 30575336

[4] Nakazawa T . Difficulty in the diagnosis of isolated immunoglobulin G4-related sclerosing cholangitis. Dig Endosc. (2019) 31:391–2. doi: 10.1111/den.13407 30920057

[5] Orozco-Gálvez O Fernández-Codina A Lanzillotta M Ebbo M Schleinitz N Culver EL . Development of an algorithm for IgG4-related disease management. Autoimmun Rev. (2023) 22:103273. doi: 10.1016/j.autrev.2023.103273 36682575

[6] Zhu M Li H Zhou W Wang W Yin Y Xu S . Relapsing immunoglobulin G4-related sclerosing cholangitis during maintenance treatment with low-dose steroids: a case report. Trans Gastroenterol hepatol. (2023) 8:22. doi: 10.21037/tgh PMC10184037 37197250

[7] Drazilova S Veseliny E Lenartova PD Drazilova D Gazda J Grgurevic I . IgG4-Related Sclerosing Cholangitis: Rarely Diagnosed, but not a Rare Disease. Can J Gastroenterol hepatol. (2021) 2021:1959832. doi: 10.1155/2021/1959832 34970512 PMC8714375

[8] Naitoh I Kamisawa T Tanaka A Nakazawa T Kubota K Takikawa H . Clinical characteristics of immunoglobulin IgG4-related sclerosing cholangitis: Comparison of cases with and without autoimmune pancreatitis in a large cohort. Dig Liver Dis. (2021) 53:1308–14. doi: 10.1016/j.dld.2021.02.009 33664004

[9] Huggett MT Culver EL Kumar M Hurst JM Rodriguez-Justo M Chapman MH . Type 1 autoimmune pancreatitis and IgG4-related sclerosing cholangitis is associated with extrapancreatic organ failure, Malignancy, and mortality in a prospective UK cohort. Am J gastroenterol. (2014) 109:1675–83. doi: 10.1038/ajg.2014.223 PMC4552254 25155229

[10] Xiao J Li G Yang G Jia C Li B . Case report: A female case of isolated IgG4-related sclerosing cholangitis mimicking cholangiocarcinoma. Medicine. (2017) 96:e6542. doi: 10.1097/MD.0000000000006542 28422840 PMC5406056

[11] Song S Jo S . Isolated mass-forming IgG4-related sclerosing cholangitis masquerading as extrahepatic cholangiocarcinoma: A case report. World J Clin cases. (2021) 9:8773–81. doi: 10.12998/wjcc.v9.i29.8773 PMC8546832 34734055

[12] Shu Y Cheng J Ye J Pan X . Isolated IgG4-related sclerosing cholangitis with normal serum IgG4 levels-A case report. Clin Case Rep. (2020) 8:2186–90. doi: 10.1002/ccr3.3083 PMC7669420 33235755

[13] Yadav KS Sali PA Mansukhani VM Shah R Jagannath P . IgG4-associated sclerosing cholangitis masquerading as hilar cholangiocarcinoma. Indian J Gastroenterol. (2016) 35:315–8. doi: 10.1007/s12664-016-0672-x 27439915

[14] Graham RP Smyrk TC Chari ST Takahashi N Zhang L . Isolated IgG4-related sclerosing cholangitis: a report of 9 cases. Hum pathol. (2014) 45:1722–9. doi: 10.1016/j.humpath.2014.04.006 24890945

[15] Bochatay L Majno P Giostra E Frossard JL . Isolated liver hilar infiltration by igG4 inflammation mimicking cholangiocarcinoma. Case Rep gastroenterol. (2016) 10:512–7. doi: 10.1159/000448989 PMC5091269 27843427

[16] Rungsakulkij N Sornmayura P Tannaphai P . Isolated IgG4-related sclerosing cholangitis misdiagnosed as Malignancy in an area with endemic cholangiocarcinoma: a case report. BMC surgery. (2017) 17:17. doi: 10.1186/s12893-017-0214-1 28202062 PMC5311850

[17] Nakazawa T Kamisawa T Okazaki K Kawa S Tazuma S Nishino T . Clinical diagnostic criteria for IgG4-related sclerosing cholangitis 2020: (Revision of the clinical diagnostic criteria for IgG4-related sclerosing cholangitis 2012). J hepato-biliary-pancreatic Sci. (2021) 28:235–42. doi: 10.1002/jhbp.913 33586343

[18] Tanaka A Tazuma S Okazaki K Nakazawa T Inui K Chiba T . Clinical features, response to treatment, and outcomes of igG4-related sclerosing cholangitis. Clin Gastroenterol hepatol. (2017) 15:920–6.e3. doi: 10.1016/j.cgh.2016.12.038 28111336

[19] Nakazawa T Naitoh I Hayashi K Okumura F Miyabe K Yoshida M . Diagnostic criteria for IgG4-related sclerosing cholangitis based on cholangiographic classification. J gastroenterol. (2012) 47:79–87. doi: 10.1007/s00535-011-0465-z 21947649

[20] Chari ST Smyrk TC Levy MJ Topazian MD Takahashi N Zhang L . Diagnosis of autoimmune pancreatitis: the Mayo Clinic experience. Clin Gastroenterol hepatol. (2006) 4:1010–6; quiz 934. doi: 10.1016/j.cgh.2006.05.017 16843735

[21] Naitoh I Nakazawa T . Classification and diagnostic criteria for igG4-related sclerosing cholangitis. Gut liver. (2022) 16:28–36. doi: 10.5009/gnl210116 34380781 PMC8761932

[22] Nakazawa T Ikeda Y Kawaguchi Y Kitagawa H Takada H Takeda Y . Isolated intrapancreatic IgG4-related sclerosing cholangitis. World J gastroenterol. (2015) 21:1334–43. doi: 10.3748/wjg.v21.i4.1334 PMC4306181 25632210

[23] Takagi Y Kubota K Takayanagi T Kurita Y Ishii K Hasegawa S . Clinical features of isolated proximal-type immunoglobulin G4-related sclerosing cholangitis. Dig Endosc. (2019) 31:422–30. doi: 10.1111/den.13320 30570170

[24] Yamamoto M Takano KI Kamekura R Suzuki C Tabeya T Murakami R . Predicting therapeutic response in IgG4-related disease based on cluster analysis. Immunol Med. (2018) 41:30–3. doi: 10.1080/09114300.2018.1451613 30938256

[25] Nakazawa T Naitoh I Hayashi K Miyabe K Simizu S Joh T . Diagnosis of IgG4-related sclerosing cholangitis. World J gastroenterol. (2013) 19:7661–70. doi: 10.3748/wjg.v19.i43.7661 PMC3837265 24282356

[26] Majumder S Mohapatra S Lennon RJ Piovezani Ramos G Postier N Gleeson FC . Rituximab maintenance therapy reduces rate of relapse of pancreaticobiliary immunoglobulin G4-related disease. Clin Gastroenterol hepatol. (2018) 16:1947–53. doi: 10.1016/j.cgh.2018.02.049 29526692

[27] Carruthers MN Topazian MD Khosroshahi A Witzig TE Wallace ZS Hart PA . Rituximab for IgG4-related disease: a prospective, open-label trial. Ann Rheum Dis. (2015) 74:1171–7. doi: 10.1136/annrheumdis-2014-206605 25667206

[28] Khosroshahi A Bloch DB Deshpande V Stone JH . Rituximab therapy leads to rapid decline of serum IgG4 levels and prompt clinical improvement in IgG4-related systemic disease. Arthritis Rheum. (2010) 62:1755–62. doi: 10.1002/art.27435 20191576

[29] Khosroshahi A Carruthers MN Deshpande V Unizony S Bloch DB Stone JH . Rituximab for the treatment of IgG4-related disease: lessons from 10 consecutive patients. Medicine. (2012) 91:57–66. doi: 10.1097/MD.0b013e3182431ef6 22210556

[30] Yamamoto M Awakawa T Takahashi H . Is rituximab effective for IgG4-related disease in the long term? Experience of cases treated with rituximab for 4 years. Ann Rheum Dis. (2015) 74:e46. doi: 10.1136/annrheumdis-2015-207625 25862615

